# Interdisciplinary clinical debriefing in the emergency department: an observational study of learning topics and outcomes

**DOI:** 10.1186/s12873-020-00370-7

**Published:** 2020-10-07

**Authors:** Andrew Coggins, Aaron De Los Santos, Ramez Zaklama, Margaret Murphy

**Affiliations:** Department of Emergency Medicine, Westmead Hospital, Hawkesbury Road, Sydney, NSW 2145 Australia

**Keywords:** Debriefing, Professional education, Training programs, Quality improvement

## Abstract

**Background:**

Defined as a ‘guided reflective learning conversation’, ‘debriefing’ is most often undertaken in small groups following healthcare simulation training. Clinical debriefing (CD) following experiences in the working environment has the potential to enhance learning and improve performance.

**Methods:**

Prior to the study, a literature review was completed resulting in a standardised approach to CD that was used for training faculty. A pilot study of CD (*n* = 10) was then performed to derive a list of discussion topics and optimise the faculty training. The resulting debriefing approach was based on the “S.T.O.P.” structure (Summarise the case; Things that went well; Opportunities for improvement; Points of action). A debriefing aid, with suggested scripting, was provided. A subsequent observational study assessed CD within 1-h of clinical events. ‘Significantly distressing’ or ‘violent’ events were excluded. Data was collected on participant characteristics, discussion topics, and team recommendations. Study forms were non-identifiable. Subsequent analysis was performed by two investigators using content analysis of the debriefing forms (*n* = 71). Discussion topics (learning points) were coded using a modified version of the Promoting Excellence and Reflective Learning in Simulation (PEARLS) framework. One month after completion of the study, ED management staff were surveyed for reports of “harm” as the result of CD.

**Results:**

During the study period, 71 CDs were recorded with a total of 506 participants. Mean debriefing length was 10.93 min (SD 5.6). Mean attendance was 7.13 (SD 3.3) participants. CD topics discussed were divided into ‘plus’ (well-done) and ‘delta’ (need to improve) groupings. 232 plus domains were recorded of which 195 (84.1%) aligned with the PEARLS debriefing framework, suggesting simulation debriefing skills may be translatable to a clinical setting. Topics discussed outside the PEARLS framework included family issues, patient outcome and environmental factors. CD reports led to preventative interventions for equipment problems and to changes in existing protocols. There were no recorded incidents of participant harm resulting from CD.

**Conclusions:**

Topics discussed in CD predominantly aligned to those commonly observed in simulation-based medical education. Collective recommendations from CD can be used as evidence for improving existing protocols and models of care.

## Background

Debriefing can be defined as a deliberate ‘*learning conversation*’ or as a ‘*guided reflection in the cycle of experiential learning*’. [1, 2] When taken from its familiar use for simulation based medical education (SBME) to clinical environments such as an Emergency Department (ED), it has been associated with observed improvements in team performance [3–5]. Furthermore, in a clinical setting, the potential benefits of debriefing can be delivered at a relatively low cost compared to a face-to-face SBME course and without a requirement to travel to a designated simulation centre. Further benefits of Clinical Debriefing (CD) may include improved interdisciplinary understanding, development of team reflexivity and recognition of latent patient safety threats providing opportunities for pre-emptive interventions [6–9]. As a result, CD is a current area of interest in the medical education literature [6, 10].

Although debriefing has potential benefits, there is also a historically perceived risk of unintended harm [11, 12]. Concerns about immediate ‘*hot’* debriefing stem from a 2002 Cochrane review. This review suggested single debriefing interventions in non-healthcare staff may be harmful [13]. The Cochrane review also suggested there is an association between debriefing and a risk of post-traumatic stress disorder (PTSD) [13]. While these concerns should be acknowledged, recent studies of CD for healthcare providers did not report harm in over 300 debriefings [14]. Moreover, there are other reports that describe new programs, which aim to improve the performance of frontline healthcare teams. Many of these programs build in approaches to ensure provider well-being and foster individual resilience [15–17].

Prior to this study, CD in our institution typically occurred on an ad-hoc basis [18]. When CD was offered, most debriefings were not structured and were either solely formative (i.e. for learning), or primarily undertaken later in an attempt to mitigate distress (i.e. for well-being) [10]. This observed tension of competing “learning” and “emotional” needs in each debriefing, may be a barrier for facilitators seeking to translate their existing simulation skills to a clinical setting. Our main research objective was to assess to what extent the content discussed overlapped with simulation. To this end, the typical content of simulation debriefings are well documented [19, 20]. However, the current literature on CD most often reflects the ‘*need for*’ debriefing or *‘how to facilitate’,* rather than what is disucssed [4, 5, 21, 22]. This study therefore addresses a gap in the literature by examining the topics and content discussed in clinical event debriefing. Our formal hypotheses were (A) *“providers involved in clinical debriefing discuss similar topics to those generated by simulated clinical events”,* and (B) *“immediate interdisciplinary debriefing using a structured approach may result in team-based learning with a low-risk of harm”.*

## Methods

### Study setting and debriefing approach

This was a prospective observational study setting conducted at an Australian tertiary referral centre between 1st January 2019 and 30th September 2019. Protocols were approved by the local Human Research Ethics Committee as a quality assurance (QA) project. The study adhered to the Australian National Statement on Ethical Conduct in Human Research*.*

A small pilot study (*n* = 10) of clinical debriefing was carried out between September and October 2018. All of the pilot cases were adult cardiac arrests. We observed that discussions in the pilot CDs appeared to align with a simulation debriefing framework known as PEARLS (Promoting Excellence and Reflective Learning in Simulation) [17]. However, a series of other discussion topics were also observed and a collated list of topics relevant to CD was tabulated (Table 1). This list was used as the reference for coding in the prospective study.

**Table 1:** Modified PEARLS content domains derived from pilot study (*n* = 10)

Simulation PEARLS Domains [19]	Debriefing Topic	Additional Pilot Study CD Domains	Debriefing Topic
1	Decision Making	8	Family / Social
2	Technical Skills	9	Bad Outcomes / Distress
3	Communication	10	Preparation / Pre-arrival factors
4	Resource Utilisation	11	Space / Equipment / Environmental
5	Leadership		
6	Situational Awareness	12	Unclassifiable Clinical Issue / Other Discussion
7	Teamwork		

**Fig. 1:**
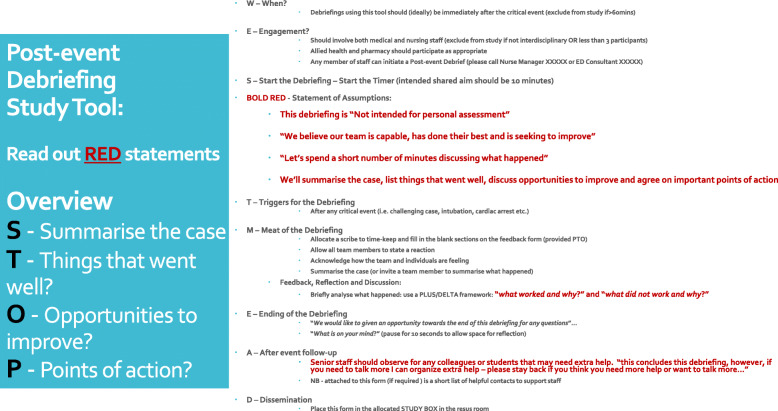
Debriefing Cognitive Aid

Further, as a result of the observations of the pilot study (supplemented by a review of the literature aided by a senior librarian) we assembled a locally appropriate blended approach to CD facilitation (Fig. 1). This CD facilitation approach blended two published debriefing models. We used the psychological safety focus from “*I.N.F.O.*” (*Immediate*, *Not* for personal assessment, *Fast* facilitated feedback, and *Opportunity* to ask questions) [23] and the structure from “*S.T.O.P.*” (*Summarise* the case; *Things* that went well; *Opportunities* for improvement; *Points* of action) [14].

This blended model was used for faculty development with 13 ED specialists, 1 social worker, 5 senior nurses and 18 ED registrars (residents) trained in CD. The total length of faculty training for debriefing was 40 min, although many of the pool of debriefers had additional prior simulation or CD training and experience [7]. Facilitators conducting debriefings were regularly reminded of the availability of scripted instructions and suggested facilitation approaches. Debriefers were asked to standardise the opening of CDs, but not given instructions on ‘what to discuss’. Prior to commencement of the study, we undertook a 4-week period of advertising of the CD project. This period orientated many permanent staff to the listed inclusion and exclusion criteria. In addition, each month during the study, a re-orientation email was sent to our regular staff, along with reminders at local team meetings.

### Inclusion criteria

The study inclusion criteria were: (1) Debriefing occurring in an acute care setting; (2) Debriefing of a clinical event; (3) Debriefing includes ≥3 persons; (4) Debriefing occurring < 1-h post event. The study exclusion criteria were: (1) Simulated event debriefings; formal ‘late’ debriefings; (2) morbidity and mortality and formal meeting discussions; (3) events involving injury to staff; (4) events associated with significant distress (i.e. an extremely distressing event).

No restrictions were placed on the clinical content of discussion or location of the event. No limits were placed on the time of day that the debriefing took place. All participants were healthcare providers or students – and the facilitators were all ED staff. All known debriefings in the study window were included but given the lack of documentation in medical records, it is possible that debriefings that occurred were not included. We mitigated this potential bias by ensuring a systematic implementation with widespread and regular communication as outlined above.

### Outcome measures and analysis

The primary outcome measure of this study was the content analysis of discussion topics in CDs against the list of domains identified in the pilot study (Table 1). Qualitative analysis, content analysis and coding were performed by a single investigator and cross-checked by a second investigator for accuracy and errors. Using consensus between two investigators (AC and ADS), topics were allocated a PEARLS domain code. Secondary outcomes included other reported data points on the audit form such as length of debriefings, time of commencement, number of staff present, designation of debriefer, designation of instigator, *‘plus’* points discussed, *‘delta’* points discussed, points of action (including further debriefing) and team recommendations. The case description was defined under six predefined headings (i.e. Trauma, Airway, Resuscitation, Psychiatric, Medical and Surgical) with a further three categories added at the conclusion of the study for appropriate coding (Thoracotomy, Obstetric and Unclassified). CD ‘harm’ was assessed 1 month after the conclusion of the study by checking with ED managers and reviewing the hospital incident management system (IMS) for ‘*reports of harm from debriefings’*.

**Fig. 2:**
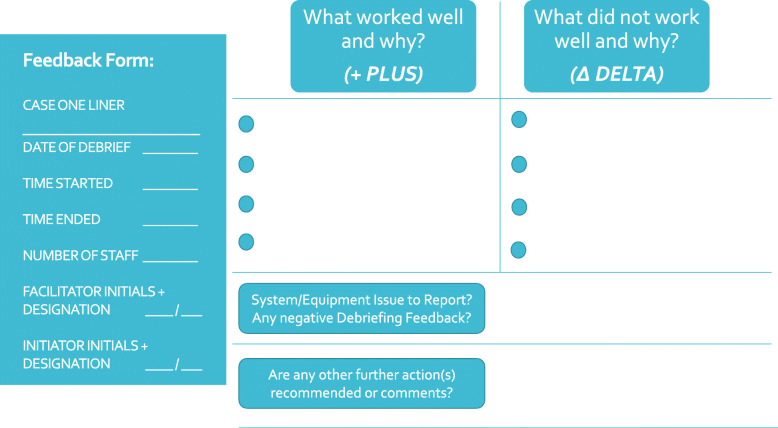
Study Data Collection Form

To reduce the risk of observer bias, an independent member of the clinical team was asked to fill in the data form (i.e. a debriefing scribe working with the CD debriefer). The data collection form (Fig. 2) was designed to be straight forward to complete for busy providers. Written instructions were provided to scribes to ensure standardisation of reporting.

## Results

A total of 76 study forms were submitted by healthcare staff during a 9-month pre-defined window. 5 forms were excluded from the final analysis (3 for debriefing >60mins after event and 2 due to forms being left blank).

Table 2 reports on the baseline characteristics of the debriefings observed. The total time of reported debriefings was 776 min with a total of 506 staff attending. The lead debriefers were drawn from medical, social work and nursing backgrounds with a total of 16 debriefers participating in all. All of the debriefers had received the standardised training on the suggested approach to debriefing (Fig. 1).

**Table 2:** Participant baseline characteristics (*n* = 71)

Characteristics	Result
Debriefings and location (n/%)^a^	71 events
	- 63 in ED (88.7%)
	- 5 in Ward (7.0%)
	- 2 in Theatres (2.8%)
	- 1 in Clinics (1.4%)
Debriefings occurring on weekdays 0800–1800 (n/%)	41 (57.7%)
Mean debriefing length (minutes/SD)	10.93 (SD 5.59)
Mean participants per debriefing (n/SD)	7.13 (SD 3.30)
Recommendations for formal delayed debriefings (n/%)	2 (2.81%)
Designation of CD facilitator (n/%)	49 Medical (69.0%)
	21 Nursing (29.5%)
	1 Social Worker (1.4%)
Designation of CD prompter (n/%)	36 Medical (50.7%)
	32 Nursing (45.1%)
	2 Social Worker (2.8%)
	1 Other (1.4%)

^a^All CDs were facilitated and prompted by Emergency Department (ED) staff

The primary outcome measure (domains discussed) is reported in Table 3. Domains discussed were divided into plus (well done) and delta (challenges). 232 *‘plus’* learning points were recorded of which 195 (84.1%) were coded to one of the PEARLS simulation debriefing domains [19]. 164 *‘delta’* learning points were observed of which 107 entries (61.5%) were coded to a PEARLS simulation-based debriefing domain [19].

**Table 3:** Discussion domains (*n* = 71)

PLUS (good or positive performance) discussion				DELTA (case changes or poor performance) discussion			
**Discussion Domain**	n	%	PEARLS versus non-PEARLS Total	**Discussion Domain**	n	%	PEARLS versus non-PEARLS Total
Decision Making^a^	40	17.24%	SIMULATION ‘PEARLS’ FRAMEWORK DISCUSSION REPORTS	Decision Making^a^	22	13.41%	SIMULATION ‘PEARLS’ FRAMEWORK DISCUSSION REPORTS
Technical Skills^a^	29	12.50%		Technical Skills^a^	24	14.63%	
Communication^a^	33	14.22%		Communication^a^	22	13.41%	
Resource Utilisation^a^	26	11.21%		Resource Utilisation^a^	18	10.98%	
Leadership^a^	20	8.62%	Total 195 (84.05%)	Leadership^a^	7	4.27%	Total 107 (65.24%)
Situational Awareness^a^	10	4.31%		Situational Awareness^a^	9	5.49%	
Teamwork^a^	37	15.95%		Teamwork^a^	5	3.05%	
Family / Social	2	0.86%	NON-SIMULATION FRAMEWORK DISCUSSION REPORTS	Family / Social	7	4.27%	NON-SIMULATION FRAMEWORK DISCUSSION REPORTS
Bad Outcome / Distress	4	1.72%		Bad Outcome / Distress	11	6.71%	
Preparation / Pre-arrival	21	9.05%	Total 37 (15.95%)	Preparation / Pre-arrival	5	3.05%	Total 57 (34.76%)
Space / Equipment / Environmental	6	2.59%		Space / Equipment / Environmental	21	12.8%	
Unclassified / Other	4	1.72%		Unclassified / Other	13	7.93%	
TOTAL	100%		232	TOTAL	100%		164

^a^Promoting Excellence and Reflective Learning in *Simulation* (*PEARLS)* [19]*.*

Table 4 provides a supplementary overview of the debriefings reported in terms of specific discussion topics. A broad variety of clinical cases were discussed during debriefings of which cardiac arrest (31%), medical emergencies (19.7%), airway (19.7%) and trauma (18.3%) were the most prevalent.

**Table 4:** Characteristics of cases (*n* = 71)

Clinical event type	Number of debriefings	No. participants (μ)	Length (μ / minutes)
Major Trauma (n/%)	13 (18.3%)	11.0 (SD 6.42)	6.7 (SD 3.22)
Airway (n/%)	14 (19.7%)	10.1 (SD 4.03)	6.4 (SD 1.34)
Cardiac Arrest (n/%)	22 (31.0%)	11.0 (SD 4.05)	8.4 (SD 3.75)
Psychiatric Emergency (n/%)	2 (2.8%)	6.5 (−)	9 (−)
Medical Emergency (n/%)	14 (19.7%)	11.9 (SD 8.79)	5.5 (SD 1.61)
Surgical Emergency (n/%)	1 (1.4%)	10 (−)	7 (−)
Thoracotomy (n/%)	1 (1.4%)	15 (−)	5 (−)
Obstetric (n/%)	4 (5.6%)	11.5 (SD 5)	9.3 (7.85)
Other / Unclassified (n/%)	0 (0%)	0 (−)	0 (−)

In the comments section of the data collection form, 37/71 teams documented that the clinical debriefing was useful, and participants anecdotally reported that the CD experience was positive. Table 5 summarises the quality improvement reporting generated from CDs. The reports were managed by investigators on a fortnightly basis. Team reporting from CD led to practice changes that are detailed in Table 5.

**Table 5:** Quality assurance reporting from debriefings (*n* = 49)

Debriefing Report Type	Total number of relevant reports	Example(s) of group recommendation	Documented Practice Changes	Potential Outcomes
Equipment failure or deficit reported	20 (40.8%)	End-tidal Co2 not routinely available for transport of intubated patients	EMMA™ end tidal Co2 device added to transport packs	Redundancy built into transfer pack for intubated patients
Targeted education required or recommended	13 (26.5%)	Inappropriately low triage category Unfamiliarity with obstetric medications	Individual feedback and education by mentor Shortcuts available for rarely used medications	Reduced future risk of ‘*undertriage*” and increased team familiarity with medications
Breach in standard operating procedure(s) or protocol(s)	2 (4.1%)	Use of a LUCAS-3™ compression device (contraindicated in trauma)	Laminated guidelines attached to storage area and mechanical CPR device	Reduce risk of inappropriate use of devices in future cases
Further debriefing opportunities organised	2 (4.1%)	(Poor outcome (a premature neonate died in ED), noise level was a concern to some team members	Identified need to for formal emotional debriefing	Additional debriefs to provide psychological support for affected staff
Other(s)	12 (24.5%)	Massive Transfusion Protocol (MTP) unavailable on arrival	Patient medical record number and blood available pre-arrival	Reduce risk of MTP being delayed in future cases

## Discussion

Historically, debriefing occurs after simulated events and is considered to be a time for individual reactions, team reflection, shared learning and discussion [24, 25]. Similarly, rich opportunities for learning also exist, albeit less predictably, in the real life clinical environment [26]. Previous studies report CD rates after resuscitation of between 6 and 31% [5]. In this study only 22/68 (31%) of our reported cardiac arrests were debriefed. These findings highlight a potential opportunity missed in our current approaches to clinical education. In addition, the literature suggests that while CD is desirable and feasible in healthcare settings, there is little reporting on what is actually discussed [26, 27]. This study investigated a convenience sample of 71 CDs. In this discussion of the results we focus on four central topics; firstly, the study’s primary outcome of ‘*what was discussed?*’ in comparison to SBME debriefings, secondly, the effectiveness of the facilitation approaches adopted, thirdly, the local impact observed after implementation of the CD program, and finally, the potential issue of ‘harm’ associated with immediate CD.

### What content is discussed in clinical debriefings?

The PEARLS framework, often used in simulation, can be used as a universal debriefing structure [17]. In addition, PEARLS is a useful tool for facilitators to self-assess the quality and content of debriefings. The PEARLS approach aids facilitators to blend various debriefing strategies, including learner self-assessment and focused facilitation, whilst also providing a list of common topic discussion domains [19]. The PEARLS discussion domains include decision making, technical skills, communication, resource utilisation, leadership, situational awareness and teamwork. In our study we found that the majority of topics discussed during CD were in line with those described in the literature as occurring in simulation (Table 3) [19]. This is an important finding because it implies that simulation facilitation skills may be transferable to CD. In addition, the combined list of content domains presented in Table 3 may aid prospective facilitators by providing insight into the topics teams discuss during clinical debriefings [4, 19, 28].

The domains of ‘decision making’ and ‘communication’ were observed as the most common areas for positive discussions and as the most frequent area that teams would seek to improve in the future. Published evidence suggests that suboptimal communication can lead to adverse outcomes [25, 29]. Decision making errors can be magnified by the *‘framing effect’* which suggests that variance in how information is communicated, stress, workload, seniority and culture can significantly change the decisions clinical teams make [25, 30]. Strategies such as clear team structures, shared mental models and better communication have all been shown to improve the decision making of clinical teams [17, 25, 31].

In the wider context of training resuscitation teams, it is apparent that despite most staff attending face to face educational programs (e.g. Advanced Life Support), much work is needed to optimise consistency in our local teams [8, 31]. In this regard, Schmulz and Eppich (2017) describe the concept of “*team reflexivity*” amongst healthcare teams. They view healthcare teams as groups of well-trained experts that, without dedicated training, often form non-expert teams [32]. Team reflexivity describes the team’s collective ability to reflect on shared goals, processes, and outcomes of their experiences and adapt accordingly. CD may have a specific role in promoting team reflexivity, both directly from facilitated discussion, and from changes in culture resulting from routine learning conversations. To this end, in this study we observed that teamwork was commonly discussed, and evidence suggests that teamwork is pivotal in reducing healthcare errors [33, 34].

While the listed PEARLS domains covered the majority of content discussed in CDs, during the pilot study we also observed additional domains not covered by PEARLS (Table 1). These included family issues, social issues, poor outcomes, pre-arrival preparation and environmental issues. Educators leading CDs seeking to use existing SBME debriefing skills, or the PEARLS framework, should anticipate that the listed additional areas may be raised by teams, both in terms of positive and negative performance. In particular, the frequent discussion of environmental factors observed during CDs highlights the importance of the facilitator highlighting the wider applications and implications of learning during SBME activities [35, 36]. Future versions of the PEARLS framework modified for clinical environments could consider adding the additional domains mapped in our study [19].

In summary, the domains listed in Table 3 are all areas that could be discussed during CD to ensure improvements in performance and increased team reflexivity. The adoption of new educational strategies such as targeted SBME and a raised awareness of crisis resource management principles are associated with observed improvement in team performance [4, 35]. CD may have a future role in supporting existing ALS training by reinforcement of good habits, revision of prior learning and aiding translation of known best practices to a clinical setting [4–6, 36].

### What were the practical implications of the facilitation methods described?

Although reported debriefing times in this study were relatively short, the use of a cognitive aid and a structured approach appeared to assist with facilitating brief yet high-yield debriefings [23]. There is currently a wide range of well-designed feedback tools and instructional aids that address ‘how to debrief’ in various contexts [37–39]. The approaches described in the literature include ‘INFO’, ‘PEARLS’, ‘TALK’ ‘TEAMSTEPPS’ and ‘TeamGAINS’ [14, 39–43]. There is, however, no universally applicable clinical debriefing method. All methods listed have pros and cons and should be applied wisely, with consideration given to local historical, clinical and cultural context.

In addition, there is a paucity of evidence on the optimal length of CD with systematic reviews reporting length of debriefings ranging from 2 to 30 min [44]. In this study, the CDs (mean length 10.93 min) appeared to foster learning in a typically time constrained Emergency Department environment. These findings are consistent with previous studies of a structured CD where numerous topics were addressed within a ten-minute timeframe [14, 22].

Despite the limitation of our study being conducted at a single centre, reports of similar programs being implemented successfully suggest that CD is feasible in busy clinical settings. Furthermore, despite the short reported CD lengths, all staff and students involved in debriefings were given the opportunity to ask questions or seek further follow-up as part of the “*points of action*” heading [23]. For example, the facilitator sharing links to the correct local protocols for similar future events may be all that is required in regards to closing the loop.

In the reported literature, although active team leaders commonly take charge of post-event debriefings, their busy role during the case may potentially bias or inhibit their ability to effectively faciliate [4, 34]. To mitigate this, it has been suggested that a less active member within the team or external provider facilitate discussions [4, 14]. This alternative approach could provide an opportunity for multiple members of the interdisciplinary team to both instigate and deliver CD [23]. Our study reveals that, while the majority of CDs were led by medical staff (Table 2), more than half of CDs were prompted by other members of the interdisciplinary team. We believe that this encouraging finding highlights the importance of interdisciplinary involvement, for the successful implementation and ongoing sustainability of a structured CD program [14].

### What local changes were observed following implementation of the debriefing program?

The practicalities and ergonomics of the resuscitation environment have a significant impact on performance [43]. Further, standardised operating procedures (SOP) significantly influence how a team delivers emergency care [17]. This is relevant to CD because errors in the application of SOPs may be identified by experienced teams familiar with the work on the frontline. In this study we found a number of examples of errors in existing SOPs and potential latent safety threats (Table 5). We acted to resolve the various issues arising. Assuming that confidentiality is maintained, CD has the potential to provide useful quality assurance information and allow for pre-emptive actions to avoid adverse outcomes. This is a topic that could be explored in more detail with studies that analyse how CD can be used as a quality reporting tool.

In this study, significant changes in practice resulted from points flagged during CD, including the redesign of the paediatric arrest trolley, the availability of end-tidal CO2 monitoring for transferring intubated patients, and blood to resuscitate the exsanguinating trauma patient. As the data collection of each debriefing was non-identifiable, groups of learners may have been more likely to feel confident that they could speak up safely in relation to which system factors should change [6].

In summary, clinical environments that are well designed and align with the needs of teams, facilitate optimal management of critically unwell patients [34, 45]. In this study, providers were asked by each debriefer whether they wanted to report system issues. These questions led to a series of pragmatic learning points which appeared to enhance team-based learning and, through reporting (Table 4), the wider patient safety needs of the institution [8].

### Does clinical debriefing cause harm?

In regard to harm associated with CD, while equipoise remains around the negative effects of compulsory debriefing in lay people, healthcare staff in this study reported that their debriefing experience was anecdotally positive [12]. In this study all team members were given an ‘opt out’ if desired, and where applicable, opportunity to speak during the debriefing. The *‘points of action’* section ended the debriefing and included the offer of further support if required [23]. Educators leading the program and clinical managers observed for adverse events associated with CD. However, we are not aware of any reported incidences of staff seeking further assistance from our faculty or in-house psychological support services as the result of a negative CD experiences [16, 46].

It is our view, based on the results of this study and the wider literature, that CD is likely to produce positive outcomes including an increase in team performance, but that implementation is a key consideration to ensure success [47]. While healthcare staff are likely to be relatively resilient in the face of challenging situations [44], the potential negative effects on both psychological safety, team culture and individuals (including burnout) require further study in a range of clinical environments [46, 48, 49]. Furthermore, we acknowledge that unintended negative consequences and staff dissatisfaction are a risk if CD programs are implemented poorly [16, 44].

### Limitations

This study reports on indirect observation of debriefing practices at a single institution, so caution must be used in extrapolating the results. Various forms of bias may have compromised our results, notably the Hawthorne effect may have changed behaviour of our facilitators. Furthermore, we note there are limitations of using forms to report the subtleties of a typical debriefing conversation. Therefore, we acknowledge that some of our conclusions should be considered anecdotal. Finally, the local culture and the risk of harm, highlighted in other studies, should always be considered when conducting any form of debriefing. To this end, in keeping with contemporary studies of CD, while no harm was reported in this study, the long term consequences of clinical debriefing remain uncertain.

## Conclusion

Facilitation of CD is an emerging skill for frontline clinical educators and the wider simulation educator community, who may be increasingly asked to use their skills in clinical settings. Results from this study suggest that many of the skills typically used in simulation debriefing overlap with those required for CD. Implementation of a CD program using a structured approach appears to be feasible when supported by faculty development and interdisciplinary engagement. In addition, CD has the potential to provide useful quality improvement insights from frontline healthcare workers.

## Data Availability

No additional data is available. Components of the presented debriefing dataset are available from the authors on request.

## Abbreviations

CDs: Clinical debriefings
S.T.O.P.: Summarise the case; Things that went well; Opportunities for improvement; Points of action
PEARLS: Promoting Excellence and Reflective Learning in Simulation
SBME: Simulation Based Medical Education
PTSD: Post-Traumatic Stress Disorder
HREC: Human Research Ethics Committee
ED: Emergency Department
I.N.F.O.: Immediate; Not for personal assessment; Fast facilitated feedback; Opportunity to ask questions
MTP: Massive Transfusion Protocol
LUCAS-3: Lund University Compression Assist Device
SOP: Standardised Operating Procedure
